# Behavioural intention of receiving COVID-19 vaccination, social media exposures and peer discussions in China

**DOI:** 10.1017/S0950268821000947

**Published:** 2021-04-23

**Authors:** Sitong Luo, Meiqi Xin, Suhua Wang, Junfeng Zhao, Guohua Zhang, Lijuan Li, Liping Li, Joseph Tak-fai Lau

**Affiliations:** 1 Centre for Health Behaviours Research, JC School of Public Health and Primary Care, The Chinese University of Hong Kong, New Territory, Hong Kong SAR, China; 2 Graduate School of Baotou Medical College, Baotou Medical College, Baotou, Inner Mongolia, China; 3 Department of Psychology, School of Education, Henan University, Kaifeng, China; 4 Department of Psychology, School of Psychiatry, Wenzhou Medical University, Wenzhou, China; 5 School of Public Health, Dali University, Dali, Yunnan, China; 6 Shantou University Medical College, Shantou, China

**Keywords:** Behavioural intention, COVID-19 vaccination, discussion, information sufficiency, social media

## Abstract

The study aimed to investigate behavioural intentions to receive free and self-paid COVID-19 vaccinations (BICV-F and BICV-SP) among Chinese university students if the vaccine was 80% effective with rare mild side effects, to examine their associations with social media exposures and peer discussions regarding COVID-19 vaccination, and to explore the mediational role of perceived information sufficiency about COVID-19 vaccination. An online anonymous survey (*N* = 6922) was conducted in November 2020 in five Chinese provinces. Logistic regression and path analysis were adopted. The prevalence of BICV-F and BICV-SP were 78.1% and 57.7%. BICV-F was positively associated with the frequencies of passive social media exposure (adjusted odds ratio (AOR) = 1.32, *P* < 0.001), active social media interaction (AOR = 1.13, *P* < 0.001) and peer discussions (AOR = 1.17, *P* < 0.001). Indirect effects of the three factors on BICV-F via perceived information sufficiency were all significant (*P* < 0.001). The direct effect of active social media interaction on BICV-F was significantly negative (*P* < 0.001). Similar associations/mediations were observed for BICV-SP. The COVID-19 vaccination intention of Chinese university students needs improvement. Boosting social media exposures and peer discussions may raise students' perceived information sufficiency and subsequently increase their vaccination intention. Considering the potential negative effect of active social media interaction, caution is needed when using social media to promote COVID-19 vaccination.

## Introduction

Immunisation is seen as the most promising measure to end the COVID-19 pandemic [1]. Despite the remarkable progress of COVID-19 vaccine development, vaccine hesitancy has raised public health concerns [2]. Prior to the COVID-19 pandemic, vaccine hesitancy was listed as one of the top 10 threats to global health by the World Health Organization (WHO) given the resurgence of vaccine-preventable diseases [3]. A number of studies conducted across countries (e.g. France, Italy, U.S.A., Canada, Israel, China and Indonesia) have warned that vaccine hesitancy may undermine the success of future COVID-19 vaccination programmes, with the reported prevalence of acceptability or behavioural intention of receiving COVID-19 vaccination ranging from 57.6% to 93.3% [4–10]. It is warranted to understand the facilitators and barriers that influence the acceptance of COVID-19 vaccines.

Social media are the major platforms for information seeking and discussion about COVID-19 [11, 12]. As previous studies have shown that online information influenced people's perceptions, attitudes and intentions regarding vaccination (e.g. HPV vaccination) [13–15], COVID-19 vaccination-related information on social media is potentially important in shaping the public's intention to vaccinate. There is a dearth of empirical studies looking at such relationships. It is common for people to access professional information about vaccines' safety and efficacy via social media [16]. Correct information on vaccination may strengthen their confidence and trust of vaccines [17]. For instance, previous studies on influenza and HPV vaccinations illustrated that the frequency of exposure to vaccination-related information via social media was positively associated with positive beliefs and utilisation of those vaccines [18, 19]. However, social media sometimes convey misinformation and anti-vaccination sentiments, such as conspiracy theories, exaggerated side effects and down-graded vaccine efficacy that may increase vaccine hesitancy [20, 21]. Examples include those cases involving HPV and MMR (measles, mumps and rubella) vaccinations [18, 22]. Warnings about such potential negative impacts on COVID-19 vaccination via social media have been discussed [20, 23]. A number of influential international health organisations have partnered with social media giants to combat COVID-19 and promote COVID-19 vaccination. For instance, some platforms (e.g. Facebook) have been directing their users to webpages of the WHO while they are seeking information about COVID-19 vaccines, so as to provide them with accurate and reliable information and remove false claims about COVID-19 vaccines that have been debunked by the WHO [24, 25].

Along with social media influences, peer discussion may affect individuals' intention to vaccinate against COVID-19, as it is another major source of information and social influences which takes place both in online and offline settings [26]. A U.S. survey reported that 38% of the general population indicated that their family members' and friends' opinions would influence their COVID-19 vaccination decisions [27]. Peer communication may contribute to the creation of social norms that may enhance the intention to vaccinate [28]. A qualitative Singaporean study reported that motivation regarding influenza vaccination was strengthened via communications with friends [29]. In contrast, peer discussion that involves negative attitudes about vaccines may diminish the intention to vaccinate [28]. To the best of our knowledge, no study has examined the association between the frequency of peer discussions about COVID-19 vaccines and the intention of receiving COVID-19 vaccination.

People may feel inadequately informed about the expedited COVID-19 vaccine development, whereas the perceived sufficiency of the obtained information is important for making decisions of receiving vaccination [30]. Perceived sufficiency of the information related to vaccination was positively associated with influenza vaccination and negatively associated with confusion and mistrust of influenza vaccines [31, 32]. The availability of sufficient information was also associated with a lower level of parental scepticism and hesitancy of early childhood vaccination [33]. However, sufficient information about the negative aspects of COVID-19 vaccines may deter people from vaccination. For instance, parents who rejected childhood vaccinations had searched for a large amount of information about vaccination [34]. Moreover, perceived information sufficiency may mediate between the frequencies of social media exposures/peer discussions about COVID-19 vaccines and the intention of receiving COVID-19 vaccination, as such exposures/discussions may increase perceived sufficiency of related information, which may in turn boost or hinder the vaccination intention. No study has looked at such mediations.

A few studies have examined the intention of receiving COVID-19 vaccination among university students in Europe and Southeast Asia [35–37]. COVID-19 vaccination among university students is important as the campus setting often involves close contacts and high vulnerability of COVID-19. It is also a good setting for health promotion, which has been used effectively in promoting HPV vaccination [38] and flu vaccination [39]. COVID-19 vaccination among university students may avoid closures of campuses that may affect learning. Furthermore, university students are intensive social media users who often use social media as the main source of information seeking, sharing and exchanges [40]. It is thus warranted to investigate whether the information obtained from social media and peer discussions about COVID-19 vaccination would increase or decrease the vaccination intention among university students.

The current study investigated the prevalence and associated factors of behavioural intentions of receiving COVID-19 vaccination among Chinese university students if the vaccine was 80% effective with rare mild side effects. The factors included (1) the frequency of passive exposure to information about COVID-19 vaccination on social media, (2) the frequency of active interaction about COVID-19 vaccination on social media, (3) the frequency of peer discussions about COVID-19 vaccination and (4) the level of perceived sufficiency of the obtained information about COVID-19 vaccination for decision-making. As the aforementioned associations could both be positive or negative, the four alternate hypotheses were two-sided. In addition, a mediation hypothesis was tested, that the associations between the frequencies of passive social media exposure/active social media interaction/peer discussions and the vaccination intention would be fully or partially mediated by perceived information sufficiency about COVID-19 vaccination.

## Methods

### Study design

An anonymous cross-sectional survey was conducted during 1–28 November 2020 among university students in China via an online survey platform. Through personal networks, a number of academic staff of five universities in five provinces of mainland China formed a fieldwork team. The five provinces (Zhejiang in the east, Yunnan in the south-west, Guangdong in the south, Inner Mongolia in the north and Henan in the middle) represented to some extent the country's geographical and socioeconomic variations. The researchers selected 165 classes of various grades (e.g. year 1 to 4) within the faculties of arts, sciences, social sciences, economics or management, engineering and medicine or pharmacy (and others) of the five participating universities by convenience sampling. The teachers or student representatives of the selected classes helped to send an invitation message, a QR code to access the online questionnaire and three to five daily reminders of participation to all the students of the selected classes via the existing class-based WeChat groups used for class administration. WeChat has over 1 billion Chinese subscribers and involves multiple functions (e.g. messaging, sharing news/photos/documents and digital payment).

The inclusion criteria included being (a) full-time students of the selected universities and (b) able to read and write Chinese. The exclusion criterion was a self-reported experience of having received experimental COVID-19 vaccination by the time of the survey (*n* = 18). The questionnaire was self-administered and took about 10–15 min to complete. In the invitation message sent to the students and at the beginning of the online questionnaire, the aims, procedures and voluntary and anonymous nature of the study were clearly presented, and it was also stated that the submission of the questionnaire implied informed consent to participate in the survey. The students were thus able to make decisions of participation with a full understanding of the survey. Upon completion, the participant could join a lottery which offered eight prizes of 10–50 RMB (about 1.5–7.5 USD) and a symbolic ‘lucky money’ of 1 RMB for the half of the participants for each university. Ethical approval was obtained from the ethics committee of the corresponding author's affiliated university. The same method had been used in another previously published COVID-19 study targeting university students conducted by the same research team [41]. The response rate (the number of returned questionnaires divided by the number of invitations) was 72.3% (6940/9593). The final analysed sample size was 6922.

### Measures

#### Behavioural intention of receiving COVID-19 vaccination

The behavioural intention of receiving COVID-19 vaccination was assessed by two items: the participants' perceived chance of taking up (1) free and (2) self-paid (price: 400 RMB, about 60 USD) COVID-19 vaccines during the first 6 months since the vaccines' availability in the country, assuming the vaccine was 80% effective and had rare mild side effects. The response categories ranged from 1 = ‘definitely not’ to 5 = ‘definitely yes’. Those who answered ‘probably yes’ or ‘definitely yes’ were defined as having a behavioural intention of receiving COVID-19 vaccination for free (BICV-F) or having a behavioural intention of receiving COVID-19 vaccination with a self-payment (BICV-SP).

#### Exposure to information about COVID-19 vaccination via social media

*Passive social media exposure.* The participants were asked to recall their frequencies of viewing COVID-19 vaccination-related information via (1) governmental social media accounts and (2) civil social media accounts on social media platforms (e.g. Weibo and WeChat) in the past month. In China, governmental accounts and civil accounts are both accessible on major social media platforms. Some governmental agencies (e.g. the central and local governments, state media, health commissions and CDCs) use their social media accounts to communicate with the general public and disseminate official information, whereas the general public use their civil social media accounts for daily interactions. The response categories were 1 = ‘never’, 2 = ‘rarely’, 3 = ‘sometimes’, 4 = ‘often’, 5 = ‘always’. A composite scale (the frequency of passive social media exposure scale) was constructed by taking the average of the two item scores to represent the overall frequency of passive exposure (range: 1–5).

*Active social media interaction.* The participants were asked to rate their frequencies of (1) actively searching or seeking consultation about COVID-19 vaccination-related information via social media, and (2) actively sharing COVID-19 vaccination-related information with others on social media (e.g. forwarding messages, comments and ‘like’). The response categories were similar to those of the passive social media exposure. A composite scale was also constructed using the average of the two item scores to represent the overall frequency of active social media interaction (range: 1–5).

#### Peer discussions about COVID-19 vaccination

A single item, ‘How frequently did you discuss with your classmates or friends about COVID-19 vaccination in the past month?’, was used to measure the frequency of peer discussions. The responses ranged from 1 = ‘never’ to 5 = ‘always’.

#### Perceived sufficiency of the obtained information about COVID-19 vaccination for decision-making (perceived information sufficiency)

A single item, ‘Do you think that the information about COVID-19 vaccination you have currently obtained is sufficient for you to decide whether to take up COVID-19 vaccines?’, was used. The responses included 1 = ‘very insufficient’, 2 = ‘somewhat insufficient’, 3 = ‘neutral’, 4 = ‘somewhat sufficient’, 5 = ‘very sufficient’.

#### Background information

Background information including age, sex, ethnicity, faculty, grade and COVID-19-related experiences (i.e. whether had been mandatorily quarantined due to COVID-19 and whether had been diagnosed with COVID-19) were collected.

### Data analysis

**Fig. 1.**
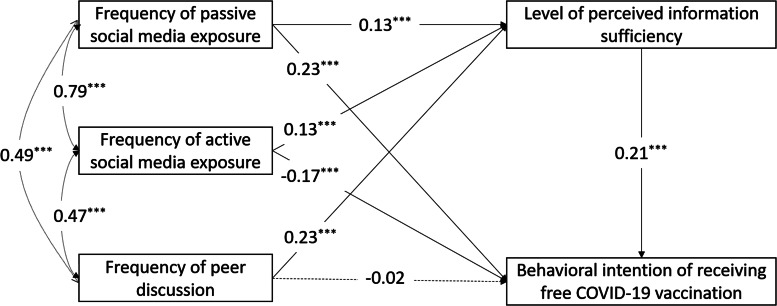
Proposed mediation model for the behavioural intention of receiving free COVID-19 vaccination. ***P < 0.001, **P < 0.01, *P < 0.05.

**Fig. 2.**
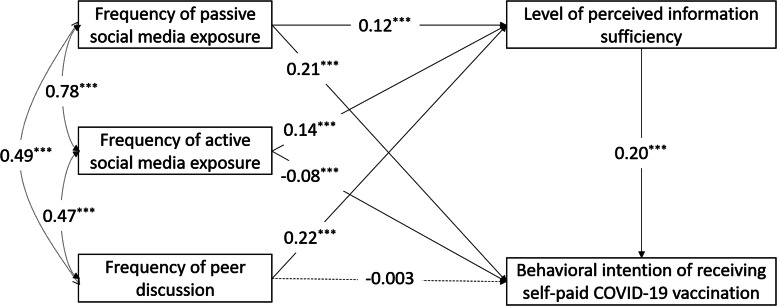
Proposed mediation model for the behavioural intention of receiving self-paid COVID-19 vaccination. ***P < 0.001, **P < 0.01, *P < 0.05.

Univariate logistic regression was conducted to examine the crude associations between the background variables/studied independent variables (i.e. frequency of passive social media exposure, frequency of active social media interaction, frequency of peer discussion, level of perceived information sufficiency) and the two binary dependent variables (i.e. BICV-F and BICV-SP). Age and the studied independent variables were included as continuous variables in the logistic regression and subsequent analyses, whereas other variables were treated as binary variables. Adjusted odds ratio (AOR) was derived to assess the association between each of the studied independent variables and the two dependent variables, adjusted for those background variables that were significantly associated with the dependent variables in the univariate analysis. Pearson's correlations among the independent variables were then derived. Finally, path analysis with weighted least square mean and variance adjusted estimation was adopted to test the hypothesised mediation models, adjusted for significant background variables (Figs 1 and 2). The direct and indirect effects of passive social media exposure/active social media interaction/peer discussions on the dependent variables via perceived information sufficiency were reported. The 95% bias-corrected confidence interval (CI) of the indirect effects were estimated based on 2000 bootstrapped samples. The paths' standardised coefficients and statistical significance were exhibited. Indices of good model fit included root mean square error of approximation (RMSEA) <0.06, comparative fit index (CFI) and Tucker Lewis index (TLI) >0.95. The analyses were conducted using SPSS 24.0 and MPlus 8.3 software. A two-sided *P* below 0.05 was considered statistically significant.

## Results

### Distributions of the studied variables

Among the 6922 participants, the mean age was 19.4 (standard deviation = 1.5); the majority were females (63.6%) and Han people (86.8%). The main faculties of the participants included arts (12.9%), science (10.2%), engineering (11.8%) and medicine/pharmacy (50.3%) (Table 1). The main grades comprised of first-year (43.2%), second-year (27.4%) and third-year (16.8%). Thus, the medical/pharmaceutical students and first-year students were over-sampled. About 13.9% of the sample reported a history of mandatory quarantine due to COVID-19, and 2.8% reported a history of COVID-19 diagnosis.

**Table 1.** Distributions of the studied variables (*N* = 6922)

Variables	*n* (%) or mean ± s.d.
*Background characteristics*	
Age (years), mean ± s.d.	19.4 ± 1.5
Sex	
Male	2520 (36.4)
Female	4402 (63.6)
Ethnicity	
Han	6009 (86.8)
Others	913 (13.2)
Faculty	
Arts	896 (12.9)
Social sciences	363 (5.2)
Economics or management	378 (5.5)
Science	703 (10.2)
Engineering	819 (11.8)
Medicine or pharmacy	3525 (50.9)
Others	238 (3.4)
Grade	
First-year	2993 (43.2)
Second-year	1894 (27.4)
Third-year	1164 (16.8)
Fourth-year	562 (8.1)
Fifth-year	214 (3.1)
Master or above	95 (1.3)
History of being mandatorily quarantined due to COVID-19	
Yes	948 (13.7)
No	5974 (86.3)
History of being diagnosed with COVID-19	
Yes	194 (2.8)
No	6728 (97.2)
*Behavioural intention of receiving COVID-19 vaccination*	
If the vaccine was free of charge	
Probably yes/definitely yes	5404 (78.1)
Others (neutral/probably no/definitely no)	1518 (21.9)
If the vaccine would charge 400 RMB	
Probably yes/definitely yes	3995 (57.7)
Others (neutral/probably no/definitely no)	2927 (42.3)
*Social media exposures and peer discussions about COVID-19 vaccination*	
Frequency of passive social media exposure^a^	
Via governmental social media accounts, mean ± s.d.	2.8 ± 1.1
Via civil social media accounts, mean ± s.d.	2.7 ± 1.0
*Composite score, mean* *±* s.d.	2.7 ± 1.0
Frequency of active social media interaction^a^	
Searching or consulting, mean ± s.d.	2.5 ± 1.1
Sharing, mean ± s.d.	2.1 ± 1.2
*Composite score, mean* *±* s.d.	2.3 ± 1.0
Frequency of discussion with friends/schoolmates in the past month^a^, mean ± s.d.	2.4 ± 0.9
Level of perceived information sufficiency about COVID-19 vaccination^b^, mean ± s.d.	2.7 ± 0.9

s.d., standard deviation; COVID-19, coronavirus disease 2019.

a The response categories were 1 = ‘never’, 2 = ‘rarely’, 3 = ‘sometimes’, 4 = ‘often’, 5 = ‘always’.

b The response categories were 1 = ‘very insufficient’, 2 = ‘somewhat insufficient’, 3 = ‘neutral’, 4 = ‘somewhat sufficient’, 5 = ‘very sufficient’.

The prevalence of BICV-F and a BICV-SP was 78.1% and 57.7%, respectively. The mean level of the frequency of passive social media exposure was 2.7 (s.d. = 1.0); that of the frequency of active social media interaction was 2.3 (s.d. = 1.0) and that of the frequency of peer discussions was 2.4 (s.d. = 0.9). For these variables, ‘2’ meant ‘rarely’ and ‘3’ meant ‘sometimes’. The mean level of the perceived information sufficiency variable was 2.7 (s.d. = 0.9), with ‘2’ indicating ‘somewhat insufficient’ and ‘3’ indicating ‘neutral’. Again, these independent variables were used as continuous variables in the subsequent analysis.

### Crude associations between the background variables and BICV-F/BICV-SP

The simple logistic regression showed that the significant background factors of BICV-F included continuous age (OR = 0.95, *P* = 0.013), being a male (OR = 0.86, *P* = 0.015), being a first-year student (OR = 1.17, *P* = 0.008), experience of mandatory quarantine (OR = 0.73, *P* < 0.001) and a COVID-19 diagnosis (OR = 0.30, *P* < 0.001) (Table 2). These variables were similarly associated with BICV-SP except that there was no significant sex difference.

**Table 2.** Crude associations between the background variables and the behavioural intention of receiving COVID-19 vaccination (*N* = 6922)

Background variables	Free vaccination^a^			Self-paid vaccination^a^		
	OR	95% CI	*P*	OR	95% CI	*P*
Age (years)	0.95	0.92–0.99	0.013	0.95	0.92–0.98	<0.001
Sex (male *vs.* female)	0.86	0.77–0.97	0.015	0.92	0.84–1.02	0.112
Ethnicity (Han *vs.* others)	1.17	0.99–1.38	0.062	0.92	0.80–1.06	0.248
Faculty (medicine/pharmacy *vs.* others)	0.93	0.83–1.04	0.223	1.04	0.95–1.14	0.420
Grade (first-year *vs.* others)	1.17	1.041.31	0.008	1.33	1.21–1.47	<0.001
History of being mandatorily quarantined (yes *vs.* no)	0.73	0.63–0.86	<0.001	0.83	0.72–0.95	0.007
History of being diagnosed with COVID-19 (yes *vs.* no)	0.30	0.24–0.40	<0.001	0.35	0.26–0.47	<0.001

OR, odds ratio; CI, confidence interval; COVID-19, coronavirus disease 2019.

a Simple logistic regression on the binary intention of receiving free/self-paid COVID-19 vaccination was performed for each background variable.

### Associations between the independent variables and BICV-F/BICV-SP

Both the simple and multivariable (adjusting for the background variables) logistic regressions found that BICV-F was all positively associated with the frequency of passive social media exposure (AOR = 1.32, *P* < 0.001), the frequency of active social media interaction (AOR = 1.13, *P* < 0.001), the frequency of peer discussions (AOR = 1.17, *P* < 0.001) and the level of perceived information sufficiency (AOR = 1.53, *P* < 0.001) (Table 3).

**Table 3.** Crude and adjusted associations of the behavioural intention of receiving COVID-19 vaccination with social media exposures, peer discussions and perceived information sufficiency (*N* = 6922)

Independent variables	Free vaccination		Self-paid vaccination	
	OR (95% CI)^a^	AOR (95% CI)^b^	OR (95% CI)^a^	AOR (95% CI)^c^
Frequency of passive social media exposure	1.31 (1.24–1.39)***	1.32 (1.24–1.40)***	1.44 (1.37–1.51)***	1.44 (1.37–1.51)***
Frequency of active social media interaction	1.11 (1.05–1.18)***	1.13 (1.06–1.20)***	1.27 (1.21–1.34)***	1.28 (1.22–1.35)***
Frequency of peer discussions	1.18 (1.11–1.26)***	1.17 (1.10–1.25)***	1.28 (1.22–1.35)***	1.27 (1.20–1.34)***
Level of perceived information sufficiency	1.53 (1.44–1.63)***	1.53 (1.43–1.63)***	1.57 (1.49–1.65)***	1.55 (1.47–1.64)***

OR, odds ratio; AOR, adjusted odds ratio; CI, confidence interval; COVID-19, coronavirus disease 2019.

***P < 0.001.

a Simple logistic regression on the binary intention of receiving free/self-paid COVID-19 vaccination was performed for each independent variable.

b Multivariable logistic regression on the binary intention of receiving free/self-paid COVID-19 vaccines was performed for each independent variable, with age, sex, grade, history of being mandatorily quarantined due to COVID-19 and history of being diagnosed with COVID-19 being controlled for.

c Multivariable logistic regression on the binary intention of receiving free/self-paid COVID-19 vaccines was performed for each independent variable, with age, grade, history of being mandatorily quarantined due to COVID-19 and history of being diagnosed with COVID-19 being controlled for.

Similarly, BICV-SP was positively associated with the frequency of passive social media exposure (AOR = 1.44, *P* < 0.001), the frequency of active social media interaction (AOR = 1.28, *P* < 0.001), the frequency of peer discussions (AOR = 1.27, *P* < 0.001) and the level of perceived information sufficiency (AOR = 1.55, *P* < 0.001).

### Correlations among the independent variables

Significant correlations were found between the frequencies of passive social media exposure and active social media interaction (*r* = 0.79, *P* < 0.001), the frequencies of passive social media exposure and peer discussions (*r* = 0.49, *P* < 0.001) and the frequencies of active social media interaction and peer discussions (*r* = 0.47, *P* < 0.001) (Table 4). The frequencies of passive social media exposure (*r* = 0.34, *P* < 0.001), active social media interaction (*r* = 0.34, *P* < 0.001) and peer discussions (*r* = 0.36, *P* < 0.001) were all significantly associated with the level of perceived information sufficiency.

**Table 4.** Correlations between social media exposures, peer discussions and perceived information sufficiency (*N* = 6922)

Variables	Frequency of passive social media exposure	Frequency of active social media interaction	Frequency of peer discussions
Frequency of passive social media exposure	1	–	–
Frequency of active social media interaction	0.79***	1	–
Frequency of peer discussions	0.49***	0.47***	1
Level of perceived information sufficiency	0.34***	0.34***	0.36***

***P < 0.001; a value of 1 implies a perfect correlation.

### The mediation effects of perceived information sufficiency

The proposed mediation model for BICV-F fitted the study data well (CFI = 0.973; TLI = 0.952; RMSEA = 0.032). First, the three indirect effects of the frequencies of passive social media exposure (standardised coefficient = 0.03; 95% CI 0.02–0.04; *P* < 0.001)/active social media interaction (standardised coefficient = 0.03; 95% CI 0.02–0.04; *P* < 0.001)/peer discussions (standardised coefficient = 0.05; 95% CI 0.04–0.06; *P* < 0.001) on BICV-F via the level of perceived information sufficiency were all statistically significant. Second, the direct effect from the frequency of passive social media exposure to BICV-F was significantly positive (standardised coefficient = 0.23, *P* < 0.001). The direct effect from the frequency of active social media interaction to BICV-F was significantly negative (standardised coefficient = −0.17, *P* < 0.001). The direct effect from the frequency of peer discussions to BICV-F was statistically non-significant. The standardised coefficient and statistical significance of each pathway of the model are exhibited in Figure 1. The mediation model for BICV-SP also fitted the study data well (CFI = 0.983; TLI = 0.968; RMSEA = 0.028). Its indirect and direct effects were very similar to those of the BICV-F model (see Fig. 2).

## Discussion

The study found that about 3/4 and 3/5 of the sampled university students intended to take up free and self-paid COVID-19 vaccination, respectively, during the first 6 months since the vaccines' availability in China, assuming the vaccine was 80% effective and had rare mild side effects. The prevalence was higher than that of the Maltese university students (30.5%) [35] but was lower than that of the Italian university students (86.1%) [36] and Pilipino college students (81.3%) [37]. There are prompt needs for health promotion, especially if free COVID-19 vaccination was not made available to university students in China, although such a scenario is unlikely to occur. As university students are usually responsive to public health issues and relatively open to new measures [36], they are more likely to be better informed about COVID-19 vaccine development than the general public. It is contended that their prevalence of the intention to receive COVID-19 vaccination may be higher than that of the general population, which can be tested in future studies.

Attention should be given to the potential importance of perceived information sufficiency about the COVID-19 vaccines, as the variable was positively associated with the intention to vaccinate and mediated the associations between passive social media exposure/active social media interaction/peer discussions and the intention to vaccinate significantly. The findings suggest that boosting passive/active social media exposures and peer discussions may increase students' perceived sufficiency of COVID-19 vaccination-related information for decision-making, which may in turn raise their vaccination intention. Previous studies have found that sufficient knowledge was significantly and positively associated with health-related behaviours [42], including the uptake of vaccination such as influenza vaccination [31, 32] and childhood vaccination [33]. It is warranted to increase the perceived information sufficiency regarding COVID-19 vaccination among university students, as despite the fact that half of the participants were medical/pharmaceutical students, the average level of perceived information sufficiency was relatively low. It was only between ‘somewhat insufficient’ and ‘neutral’, implying the majority of the university students might not possess sufficient information to decide whether to take up COVID-19 vaccination. This is somehow understandable as none of the COVID-19 vaccines had been approved by the time of the survey (November 2020). Thus, little information about their efficacy and safety was then available.

The frequency of passive social media exposure (e.g. view) was significantly and positively associated with the two types of intention to vaccinate (free and self-paid) in the logistic regression. The path analysis gave a more elaborated picture, that besides the aforementioned mediation via perceived information sufficiency, passive social media exposure showed a significantly positive direct effect on the intention to vaccinate against COVID-19. The findings imply that even passive exposure (i.e. viewing posted information instead of searching or seeking consultations) may improve the vaccination intention. It is a limitation that we did not ask the students about the contents they viewed on social media platforms and whether the contents supported or hindered their intention to vaccinate. However, there are reasons to believe that the related social media messages tended to support COVID-19 vaccination, as research has shown that the Chinese media, including social media, in general frame the development of COVID-19 vaccines in China positively [16]. Furthermore, the country has taken some measures to tackle the spread of online misinformation about COVID-19 based on the Cybersecurity Law (2017) [43]. Social media users are encouraged to report such messages to the social media platforms and/or ask for fact-checking. Messages containing detected rumours and misinformation would be removed from the social media, and the responsible accounts may be suspended [43]. Thus, some ‘filtering’ of COVID-19 vaccination-related information on social media may have occurred in China, which may lead to the possibility that more positive than negative messages about COVID-19 vaccination have appeared on social media platforms. The disproportionately positive messages might have contributed to the observed positive association between passive social media exposure and the vaccination intention. As it was a partial instead of full mediation via perceived information sufficiency, other mediators may exist (e.g. increased cue to action according to the health belief model [44] or observational learning of the social cognitive model [45]). Such potential mechanisms require testing in future research.

Interestingly, although the frequency of active social media interaction (e.g. active search, consult, comment, forward and like) was positively associated with the intention to vaccinate against COVID-19 via an increased perceived information sufficiency, its direct effect on the intention was significantly negative. The findings thus suggest that besides the potential positive effects, active social media interaction may also have some elements that may de-motivate the intention of receiving COVID-19 vaccination. It is plausible that active searching may increase the likelihood of finding some negative news about COVID-19 vaccines that may increase vaccine hesitancy. Prior studies reported that the social media in China had exposed problems about several vaccines (e.g. influenza vaccine and childhood vaccines), and the concerns had reduced people's intention of taking up related vaccines [46, 47]. Thus, active social media interaction may both increase and reduce the intention of receiving COVID-19 vaccination. Other potential suppressors between active social media interaction and the vaccination intention (e.g. higher exposure to information doubting COVID-19 vaccines) need to be examined in future studies. The findings remind us about possible inconsistent effects of social media exposures and caution needed when using social media to promote COVID-19 vaccination.

The frequency of discussion with peers (e.g. friends and classmates) also presented a significantly positive association with the intention to vaccinate in the logistic regression, whereas its indirect effect on the vaccination intention via perceived information sufficiency was significantly positive. The findings support previous research which showed that peer discussions could be used to promote vaccination behaviours (e.g. promotion of HPV vaccines in China) [27–29, 42, 48]. Peer discussions are interactive communications occurring in offline or online settings, during which the questions and answers between peers may increase students' perceived information sufficiency about COVID-19 vaccination and subsequently promote their intention to vaccinate. The direct effect of peer discussions on the intention of receiving COVID-19 vaccination was statistically non-significant in the study. It may be because that the association between peer discussions and the vaccination intention was fully mediated by the perceived information sufficiency, or because that there existed some other mediators (e.g. subjective norm towards COVID-19 vaccination) and suppressors (e.g. negative attitudes towards COVID-19 vaccines from peers) that offset the potential positive and negative effects [28, 42].

Notably, the sampled university students' frequencies of passive/active social media exposures and peer discussions regarding COVID-19 vaccination were not particularly high (between ‘rarely’ and ‘sometimes’). It is plausible that COVID-19 vaccination has not been made available to the public in China yet. It was thus not a very ‘hot’ topic. The situation may change over time when COVID-19 vaccines appear on the market. Future interventions to promote COVID-19 vaccination among university students may consider to boost their passive social media exposure and peer discussions, so as to help them accumulate accurate information about COVID-19 vaccines [42]. For instance, first, health organisations (e.g. China CDC) and key opinion leaders may post updated information about COVID-19 vaccines on their social media accounts frequently, which have shown to be effective at raising knowledge and intention of taking up HPV vaccination [49] and childhood vaccination [50]. Second, to attract students to view the vaccination-related information on social media, live presenters and animated presentation are suggested to be used. A recent study analysed the top 100 widely viewed COVID-19 vaccine videos on YouTube, and found almost all of the videos (90%) featured a live presenter, whereas 10% featured an animated presentation [51]. Third, links to pages of COVID-19 vaccines on the websites of official health organisations (e.g. WHO and CDC) should be widely disseminated on social media platforms for accessing accurate information and fact-checking [52]. In addition, on-campus seminars/events about COVID-19 vaccination and dissemination of evidence-based materials (e.g. signs and posters) [38] are suggested to stimulate peer discussions about COVID-19 vaccination.

This study has some limitations. First, the cross-sectional design had a limited ability of causal inference. The path model was only exploratory. Longitudinal studies are needed to confirm the causal relationships. Second, selection bias might exist due to non-random sampling and non-responses. The sample was overrepresented by the first-year students (43.2%) who showed a significantly higher odd of vaccination intention in the regression analysis, suggesting an overestimation of the prevalence of BICV-F and BICV-SP. Medical and pharmaceutical students also accounted for a large proportion of the sample, who might have a higher level of perceived information sufficiency about COVID-19 vaccination. However, the variable was not significantly associated with the vaccination intention. The bias may thus not be substantial. Third, social desirability bias might also lead to an overestimation of the prevalence of BICV-F and BICV-SP. Last but not least, the generalisation of the study findings to a broader population should be made cautiously, as the study was limited to the university students in five universities in China.

In conclusion, the study demonstrated that the frequencies of passive social media exposure and peer discussions about COVID-19 vaccination were positively associated with the level of perceived information sufficiency of COVID-19 vaccination, which might subsequently increase both the intentions to receive free and self-paid COVID-19 vaccinations. Future vaccination promotion programmes targeting university students may consider increasing the levels of these factors. In addition, the frequency of active interaction on social media showed a negative direct effect on the vaccination intention. Such opposite findings remind us that exposure to social media messages may have both positive and negative effects on the vaccination intention and caution is needed when using social media platforms to promote COVID-19 vaccination. Future studies need to confirm the current study findings.

## Data Availability

The paper has contained sufficient information to allow others to understand the findings. Readers can contact the authors if they want access to materials to replicate the findings.
